# Navigated Transcranial Magnetic Stimulation Motor Mapping Usefulness in the Surgical Management of Patients Affected by Brain Tumors in Eloquent Areas: A Systematic Review and Meta-Analysis

**DOI:** 10.3389/fneur.2021.644198

**Published:** 2021-03-04

**Authors:** Giuseppe Emmanuele Umana, Gianluca Scalia, Francesca Graziano, Rosario Maugeri, Nicola Alberio, Fabio Barone, Antonio Crea, Saverio Fagone, Giuseppe Roberto Giammalva, Lara Brunasso, Roberta Costanzo, Federica Paolini, Rosa Maria Gerardi, Silvana Tumbiolo, Salvatore Cicero, Giovanni Federico Nicoletti, Domenico Gerardo Iacopino

**Affiliations:** ^1^Department of Neurosurgery, Cannizzaro Hospital, Trauma Center, Gamma Knife Center, Catania, Italy; ^2^Department of Neurosurgery, Highly Specialized Hospital and of National Importance “Garibaldi”, Catania, Italy; ^3^Department of Experimental Biomedicine and Clinical Neurosciences, School of Medicine, Postgraduate Residency Program in Neurological Surgery, Neurosurgical Clinic, AOUP “Paolo Giaccone,” Palermo, Italy; ^4^Neurosurgery Unit, Department of Clinical-Surgical, Diagnostic and Pediatric Sciences, University of Pavia, Pavia, Italy; ^5^Division of Neurosurgery, Villa Sofia Hospital, Palermo, Italy

**Keywords:** NTMs, motor mapping, surgical planning, glioma, craniotomy, tractography

## Abstract

**Background:** The surgical strategy for brain glioma has changed, shifting from tumor debulking to a more careful tumor dissection with the aim of a gross-total resection, extended beyond the contrast-enhancement MRI, including the hyperintensity on FLAIR MR images and defined as supratotal resection. It is possible to pursue this goal thanks to the refinement of several technological tools for pre and intraoperative planning including intraoperative neurophysiological monitoring (IONM), cortico-subcortical mapping, functional MRI (fMRI), navigated transcranial magnetic stimulation (nTMS), intraoperative CT or MRI (iCT, iMR), and intraoperative contrast-enhanced ultrasound. This systematic review provides an overview of the state of the art techniques in the application of nTMS and nTMS-based DTI-FT during brain tumor surgery.

**Materials and Methods:** A systematic literature review was performed according to the PRISMA statement. The authors searched the PubMed and Scopus databases until July 2020 for published articles with the following Mesh terms: *(Brain surgery OR surgery OR craniotomy) AND (brain mapping OR functional planning) AND (TMS OR transcranial magnetic stimulation OR rTMS OR repetitive transcranial stimulation)*. We only included studies regarding motor mapping in craniotomy for brain tumors, which reported data about CTS sparing.

**Results:** A total of 335 published studies were identified through the PubMed and Scopus databases. After a detailed examination of these studies, 325 were excluded from our review because of a lack of data object in this search. TMS reported an accuracy range of 0.4–14.8 mm between the APB hotspot (n1/4 8) in nTMS and DES from the DES spot; nTMS influenced the surgical indications in 34.3–68.5%.

**Conclusion:** We found that nTMS can be defined as a safe and non-invasive technique and in association with DES, fMRI, and IONM, improves brain mapping and the extent of resection favoring a better postoperative outcome.

## Introduction

The surgical strategy for brain glioma has changed dramatically throughout the years, shifting from tumor debunking with subtotal resection to a more careful tumor dissection with the aim of a gross-total resection (GTR) while sparing neurologic functions. This more aggressive strategy was demonstrated to increase survival, the actual goal of glioma surgery, and has been extended beyond the contrast-enhancement MRI, including the hyperintensity on FLAIR MR images and defined as supratotal resection (SpTR). It is possible to pursue this goal thanks to the refinement of several technological tools for pre and intraoperative planning including intraoperative neurophysiological monitoring (IONM), cortico-subcortical mapping, functional MRI (fMRI), navigated transcranial magnetic stimulation (nTMS), intraoperative CT or MRI (iCT, iMR), and intraoperative contrast-enhanced ultrasound (CEUS) (1–6). These methods not only allow more detailed preoperative planning but are effective in the evaluation of motor pathways integrity and are a valuable tool to guide tumor resection. It has been reported that, cortically, the closer the distance between the tumor and motor cortex, the greater the risk of new motor deficit, as demonstrated by lesion to activation distance (LAD) assessment in fMRI (1, 7–9). Similarly, at the subcortical stage, usually the proximity of the tumor to the corticospinal tract (CST) is related to a higher risk of motor deficits, but a great variability has also been reported (10–12). Moreover, repeated subcortical stimulation and its intensity modulation present a positive correlation for the detection of the CST (13, 14). The reliability of preoperative tractography is well-demonstrated to be consistent with subcortical stimulation for the CST location, in about 95% of cases (15), providing a marked improvement in the tractography data, which is not surgeon-dependent and has a strong clinical correlation allowing for reliable subcortical mapping associated with diffusion tensor imaging fiber-tracking (DTI FT) (16–19). This association has only been reported twice in literature, stating that it offers patient-specific analysis of the risk of deficit for lesions sited in eloquent areas, which can be avoided when keeping 8 mm from the CTS (15, 19). This systematic review provides an overview of the state of the art techniques in the application of nTMS and nTMS-based DTI-FT during brain tumor surgery.

## Materials and Methods

**Figure 1:**
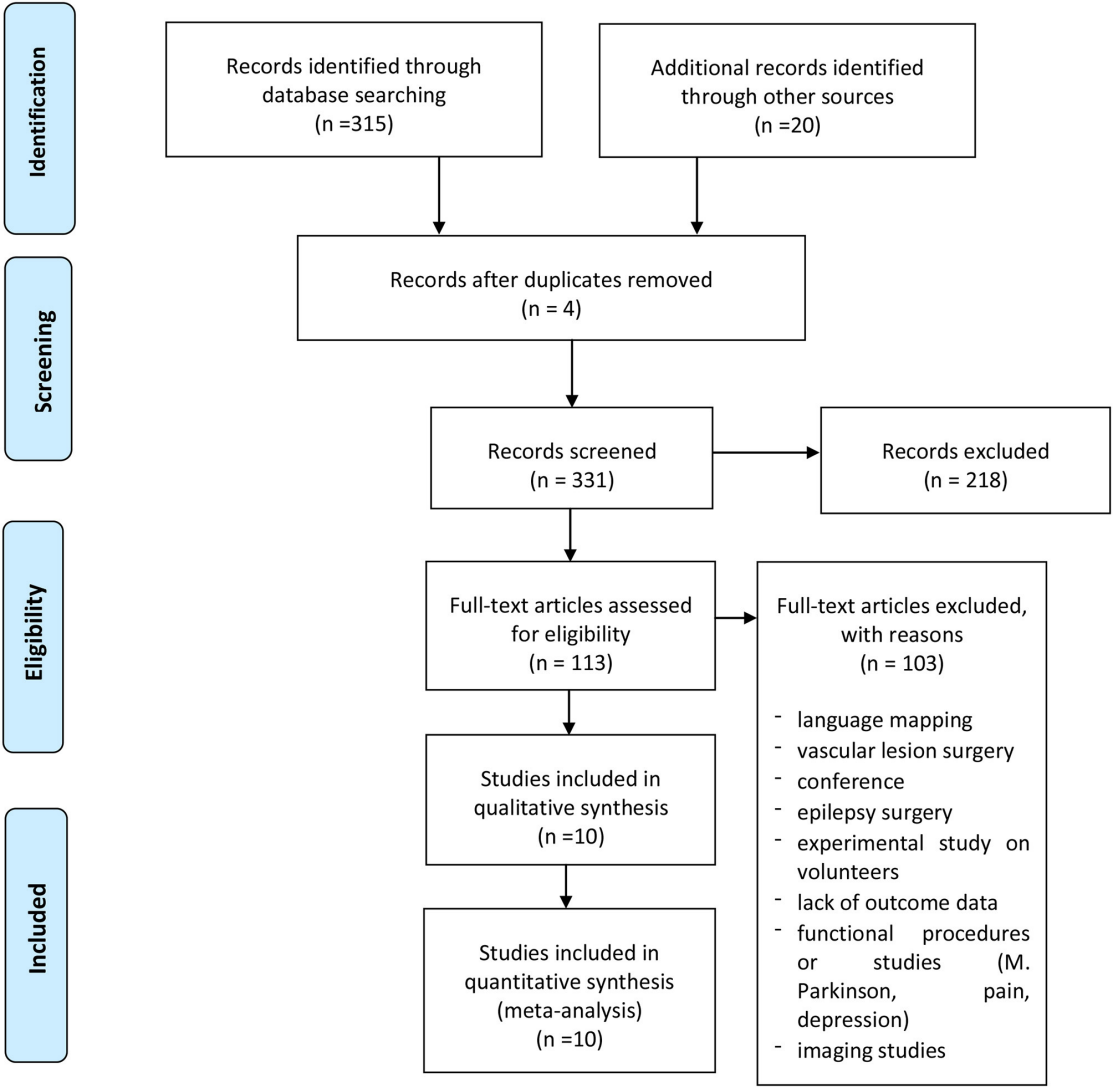
PRISMA flow diagram.

A systematic literature review was performed according to the PRISMA statement and related checklists. The authors searched the PubMed and Scopus databases until July 2020 for published articles with the following Mesh terms: *(Brain surgery OR surgery OR craniotomy) AND (brain mapping OR functional planning) AND (TMS OR transcranial magnetic stimulation OR rTMS OR repetitive transcranial stimulation)*; a language restriction to English only papers was also applied. All included studies were meticulously reviewed and scrutinized for their study design, methodology, and patient characteristics. We only included 10 studies regarding motor mapping in craniotomy for brain tumors, which reported data about CTS sparing (Figure 1). Data for all patients were recorded when available, including accuracy, GTR, STR, permanent deficits, change of strategy, and intraoperative tools used (Table 1).

**Table 1:** Summary of the systematic review including authors, motor mapping accuracy, extent of resection nTMS related, associated nTMS tools, eventual change of surgical strategy and outcome.

**Authors**	**Motor mapping accuracy**	**Extent of resection nTMS related**	**Associated nTMS tools**	**Change of surgical strategy**	**Outcome**
Paiva et al. (20)	4.1 +−1.2 mm	GTR in 33.34%; STR in 66.66%	IONM, MPRAGE MRI	Not reported	Not reported
Coburger et al. (21)	2.33 ± 0.97 mm	GTR in 85.2%; STR in 14.8%	DTI fiber tracking, fMRI, MPRAGE MRI	26.6%	Not reported
Rosenstock et al. (19)	2 mm	GTR in 50%; STR in 33%	DTI fiber tracking, IONM	Not reported	Permanent deficits in 22%
Raffa et al. (22)	<11 mm	GTR in 61.3%	DTI fiber tracking, IONM	20%	Permanent deficits in 11.4%
Jung et al. (23)	3.50 ± 0.66 mm	GTR in 75%; STR in 25%	IONM	31.5%	Permanent deficits in 5.7%
Raffa et al. (24)	1.1 + −14-8 mm	GTR in 67.6%; STR in 24.1%;	DTI fiber tracking	Not reported	Permanent deficits in 7.5%
Raffa et al. (25)	<11 mm	GTR in 73.13%; STR in 41.46%	DTI fiber tracking, IONM, sodium-fluorescein	Not reported	Permanent deficit in 9.75%
Frey et al. (26)	0.4 + −14.8 mm	GTR in 58.6%; STR in 9.4%	DTI fiber tracking, IONM	35.2%	Permanent deficits in 6.1%
Krieg et al. (27)	6.2 + −6 mm	GTR in 50%; STR in 50%	IONM, fMRI	Not reported	Permanent deficits in 12.5%
Sollmann et al. (28)	8.2 + −9.4 mm	GTR 98%	fMRI, IONM	Not reported	Permanent deficits 22%

A linear regression analysis was performed using Excel software. R2 is the coefficient of determination. We compared estimated and actual y-values, and ranges in value from 0 to 1. If it was 1, there was perfect correlation in the sample—there was no difference between the estimated y-value and the actual y-value. At the other extreme, if the coefficient of determination was 0, the regression equation was not helpful in predicting a y-value. f is the F statistic, or the F-observed value. We used the F statistic to determine whether the observed relationship between the dependent and independent variables occurred by chance (slope +− fault slope, intercepts +− fault intercepts, r2, f).

## Results

A total of 335 published studies were identified through the PubMed and Scopus databases. After a detailed examination of these studies, 325 were excluded from our review because of a lack of data object in this search, or did not report accurate data.

**Figure 2:**
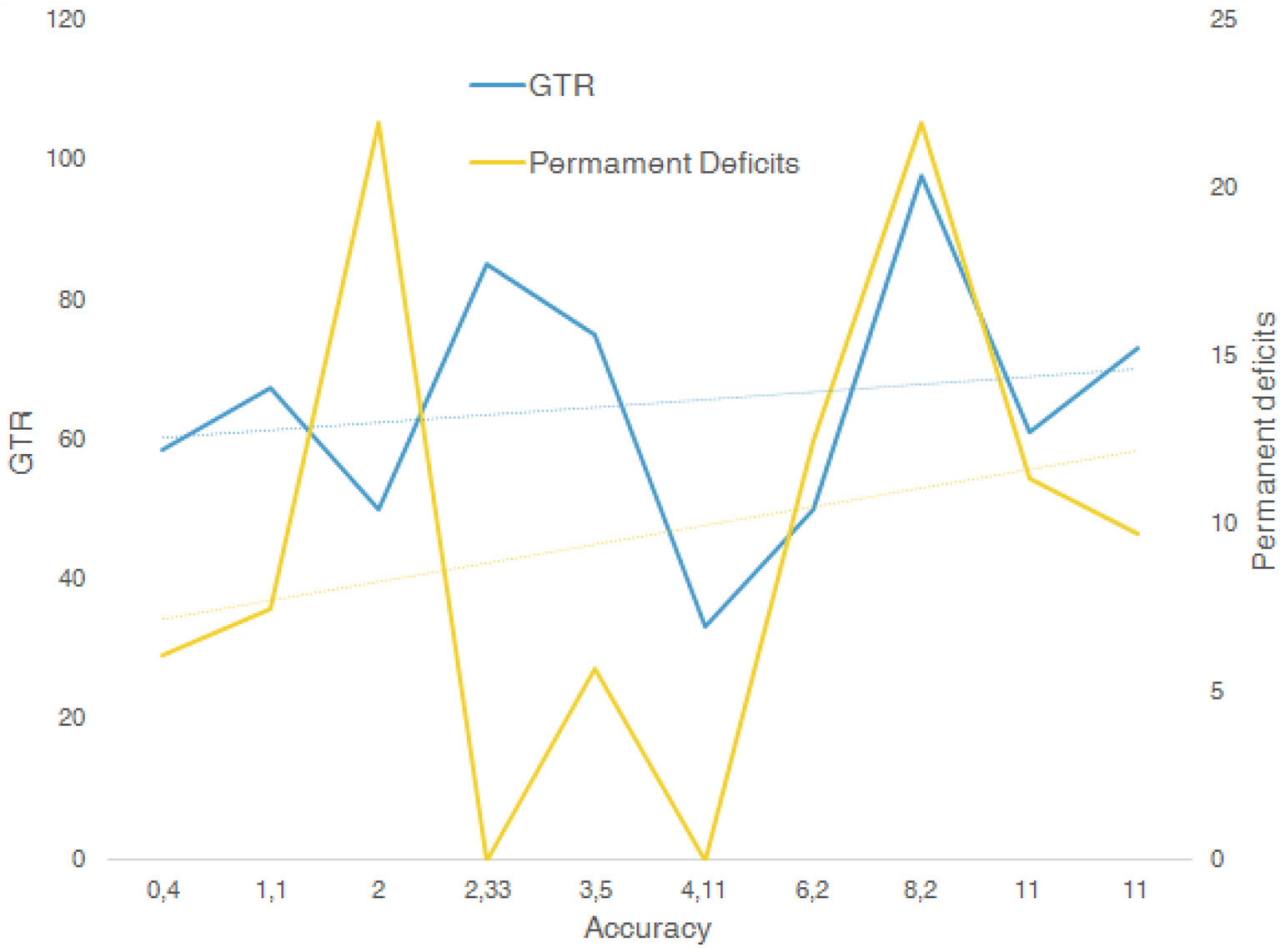
Multiple linear regression analysis between variables GTR, accuracy, and permanent deficits.

All the fits showed a low r2 value, while F was high. Linear multiple regression analysis showed that there was no correlation from the extracted data among the variables plotted in the graphs (Figure 2). Accuracy reported rate ranged from 0.4 to 14.8 mm; GTR range was 33–98%, and STR range 9.4–66.6%. The associated nTMS tools used included DTI fiber tracking, fMRI, MPRAGE MRI, IONM, and sodium-fluorescein. IONM was used in 8 out of 10 studies suggesting that this was considered the most reliable tool, followed by DTI fiber tracking (6 out of 10), fMRI (4 out of 10), and sodium fluorescence as the emerging tool (1 out of 10).

## Discussion

### Multimodal Functional Surgical Planning

The gold standard for functional assessment and surgical planning is represented by DES associated with IONM (29–32). With the aim to improve risk stratification of motor eloquent area detection in the preoperative phase, other techniques have been introduced. Function MRI is a valuable tool, which helps to obtain visuo-spatial data of motor and language functions, which can be merged with the anatomic multiplanar MRI study in navigation planning (22, 33–35). fMRI offers 59–100% sensitivity, with 0–97% specificity, which although a drawback offers a great variability operator dependent of language mapping, while tractography representation does not offer functional data (36–39). TMS mapping is not a novelty by itself, introduced in 1985 (40), it has been reported to be a valuable tool in risk stratification and the mapping of motor and language areas for surgical planning (41–50). Of notice, nTMS is used directly by neurosurgeons, in the context of the neurosurgical department and it is independent of neuroradiological availability, helping its routine use in the setting of surgical planning.

### Motor Mapping Accuracy

An important TMS parameter is stimulation focality, which corresponds to the cortical area where the TMS' electric field strength reaches half the maximum value (51, 52). The smaller this area is, the better the focally and accuracy. Thielscher and Kammer (52) reported that the variability map size documented among patients can related to different coil–cortex distances and cortex radii. The focally of a coil can be quantitatively estimated by the electric field on a hemisphere representing the brain cortex radius *r* = 8 cm. Furthermore, Thielscher and Kammer reported that the variability in map dimension among different patients is related to two parameters: coil–cortex distances and cortex radii. Thus, the variability documented in our research can be related mainly to operator-dependent variables, rather than technical TMS characteristics, confirming the reliability and the utility of nTMS in a multimodal motor mapping setting. In literature, it has been reported that TMS displayed an accuracy range of 0.4–14.8 mm between the APB hotspot (n1/4 8) in nTMS and DES from the DES spot (19, 23–28, 48, 52–57). These data endorse the reliability of nTMS in motor mapping, representing a useful tool in multimodal brain mapping. An important point is the reduction of the surgical time: nTMS plays an important role in the guidance of the intraoperative stimulation, saving time during cortical mapping. Moreover, the preoperative cortical mapping related to nTMS reduces the need of large cortical exposure, thus reducing the craniotomy size and again the surgical time related to the craniotomy opening/closing step.

### Surgical Strategy and Clinical Outcomes

nTMS reliability has been proven to be very strong and can influence the surgical indication to change from no surgery/biopsy to craniotomy removal in 34.3–68.5% of cases (23, 24, 26, 58–60). As already reported in the previous paragraph, the size of the craniotomy is reduced and thus the surgical strategy is modified according to the nTMS mapping, which allows professionals to plan for the location of the motor cortex, guiding the “no-look” positioning of the strip electrode, without direct visualization of the cortical motor cortex. Moreover, if brain mapping shows the absence of an eloquent area at the level of the anatomic cortical landmark, it allows surgeons to conduct the surgical removal through the cortex in otherwise considered functional areas. Jung et al. (23) reported a transopercular approach guided by the negative correspondence between the anatomic area and the language mapping of the nTMS, likewise another patient in which nTMS documented the absence of motor function at the level of the premised primary motor cortex in a patient affected by cavernoma, modifying the clinical management from no survey to indication of craniotomy.

In literature, several authors documented the positive influence of nTMS on surgical planning and postoperative outcome, with a significant role in risk stratification (26, 27, 31, 45, 61, 62). Interestingly, and apparently in contrast to these data, some authors reported more postoperative neurological deficits, with delayed recovery. An interpretation of this finding could be that more deficits are relative to a more aggressive surgical strategy encouraged by the combined use of DES and nTMS in eloquent areas (26). Even if in the literature there are several reports about sodium fluorescence (63, 64), it is not possible to provide statistically significant data as it is an emerging tool, reported only in 1 out of 10 of the selected paper in this review.

### Extent of Surgical Resection

About the role of nTMS and its effect on the extent of surgical resection (ESR), there are no univocal reports. Despite the fact that some authors (23) did not find a direct relation between nTMS and ESR, others documented a greater ESR in surgical series in which nTMS was associated with DES and IONM, and a longer progression-free survival (26, 27, 45, 65–67). These different findings could be related to the novelty of this technique and thus to the learning curve. Of course, a better understanding and a systematic analysis of data is required through randomized multicentric studies.

## Conclusions

From the analysis of the present systematic review, we found that nTMS can be defined as a safe and non-invasive technique, which when associated with DES, fMRI, and IONM improves brain mapping and the extent of resection with a better postoperative outcome. Of notice, the reliability of nTMS has been documented to modify the surgical strategy for oncologic patients.

## Data Availability Statement

The original contributions generated for this study are included in the article/supplementary material, further inquiries can be directed to the corresponding author/s.

## Author Contributions

GU, GS, FG, and RM: conception and design of study and analysis and/or interpretation of data. GU and GS: acquisition of data. GU, GS, FG, RM, NA, FB, AC, SF, GG, LB, RC, FP, and RG: drafting the manuscript. ST, SC, GN, and DI: revising the manuscript critically for important intellectual content. GU, GS, FG, RM, NA, FB, AC, SF, GG, LB, RC, FP, RG, ST, SC, GN, and DI: approval of the version of the manuscript to be published. All authors contributed to the article and approved the submitted version.

## Conflict of Interest

The authors declare that the research was conducted in the absence of any commercial or financial relationships that could be construed as a potential conflict of interest.

## Abbreviations

fMRI: Functional MRI
nTMS: Navigated transcranial magnetic stimulation
IONM: Intraoperative monitoring
SpTR: Supratotal resection
GTR: Gross-total resection
CST: Corticospinal tract
EOR: Extent of resection
CEUS: Contrast-enhanced ultrasound
DES: Direct electrical stimulation.
